# Supplementary figures and images for: The Smoluchowski Ensemble—Statistical Mechanics of Aggregation
Source: Entropy (Basel). 2020 Oct 20;22(10):1181. doi: 10.3390/e22101181 (PMC7597352; doi:10.3390/e22101181)

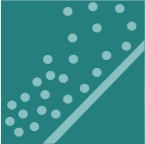

*entropy*

Supplement: Supplementary file 1 [file entropy-22-01181-s001.zip › Definitions/entropy-logo-eps-converted-to.pdf]

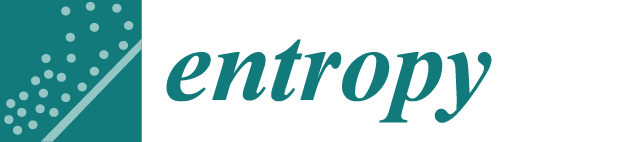

Supplement: Supplementary file 1 [file entropy-22-01181-s001.zip › Definitions/entropy-logo.png]

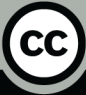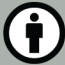

BY

Supplement: Supplementary file 1 [file entropy-22-01181-s001.zip › Definitions/logo-ccby-eps-converted-to.pdf]

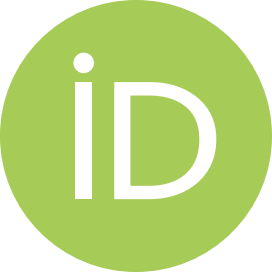

Supplement: Supplementary file 1 [file entropy-22-01181-s001.zip › Definitions/logo-orcid-eps-converted-to.pdf]

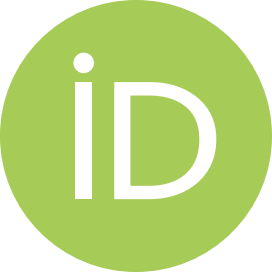

Supplement: Supplementary file 1 [file entropy-22-01181-s001.zip › Definitions/logo-orcid.pdf]

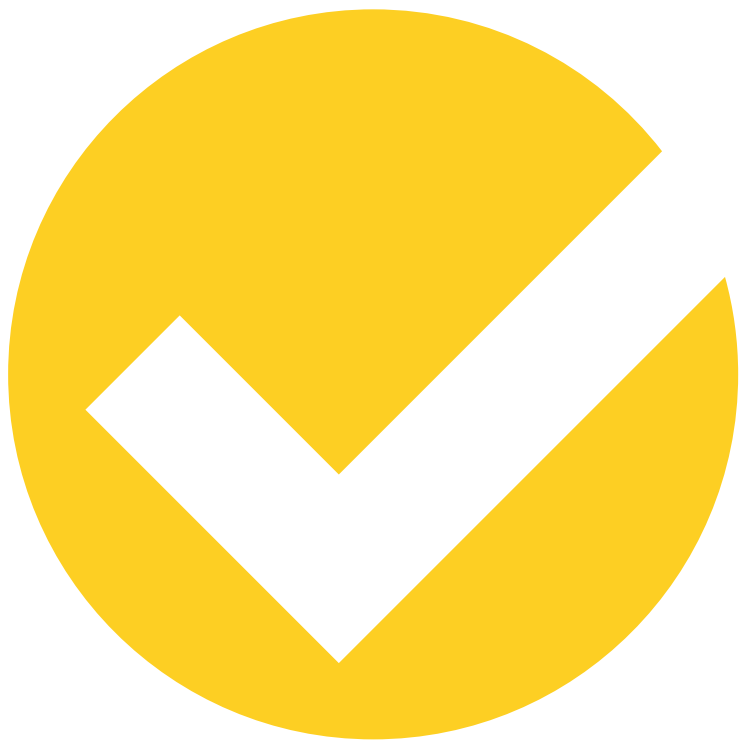

check for  
updates

Supplement: Supplementary file 1 [file entropy-22-01181-s001.zip › Definitions/logo-updates.pdf]

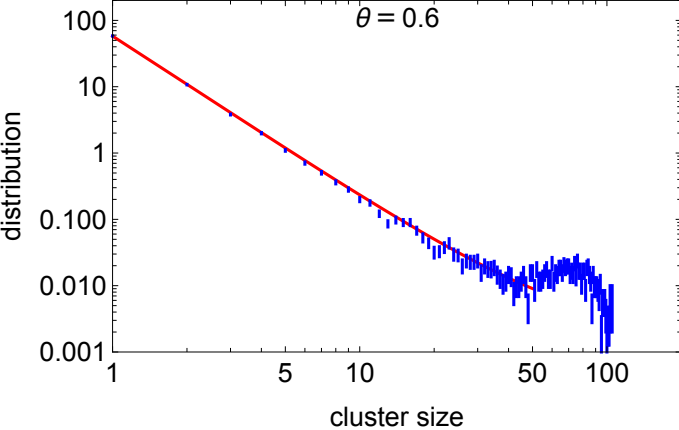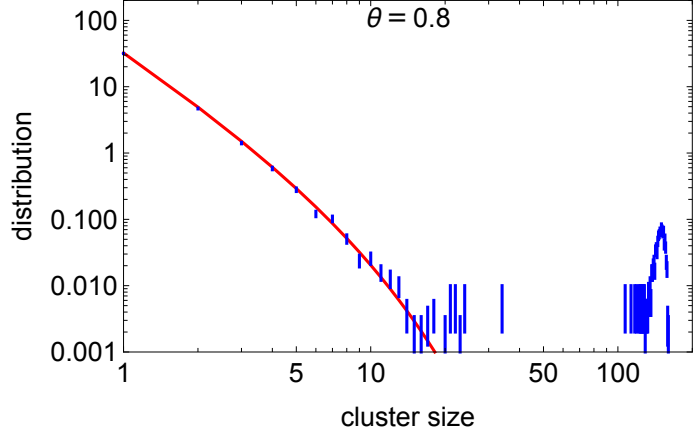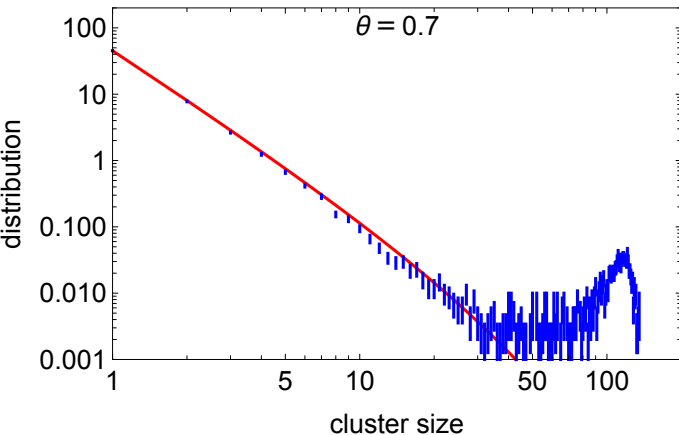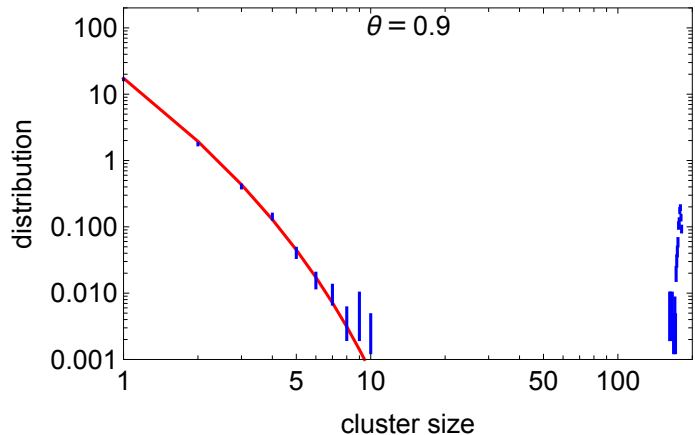

Supplement: Supplementary file 1 [file entropy-22-01181-s001.zip › fig_SM1.pdf]

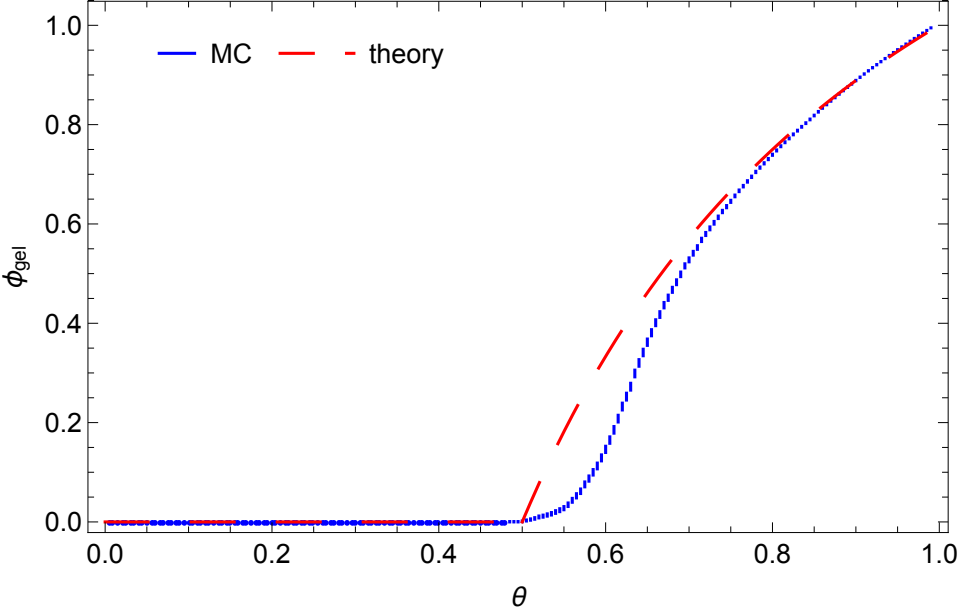

Supplement: Supplementary file 1 [file entropy-22-01181-s001.zip › fig_SM2.pdf]
